# Clinical and molecular implications of cGAS/STING signaling in checkpoint inhibitor immunotherapy

**DOI:** 10.3389/fmolb.2025.1556736

**Published:** 2025-05-16

**Authors:** Hao Chen, Yang Zhong, Rongjie Feng, Xingyu Zhu, Kang Xu, Mingjie Kuang, Wei Chong

**Affiliations:** ^1^ Clinical Research Center of Shandong University, Clinical Epidemiology Unit, School of Public Health, Cheeloo College of Medicine, Qilu Hospital of Shandong University, Jinan, Shandong, China; ^2^ Department of Epidemiology and Health Statistics, School of Public Health, Cheeloo College of Medicine, Shandong University, Jinan, Shandong, China; ^3^ Department of Orthopedics, Shandong Provincial Hospital Affiliated to Shandong First Medical University, Jinan, Shandong, China; ^4^ Department of Gastrointestinal Surgery, Shandong Provincial Hospital Affiliated to Shandong First Medical University, Key Laboratory of Engineering of Shandong Province, Jinan, Shandong, China

**Keywords:** cGAS/STING, checkpoint inhibitors therapy, tumor immunogenomics, predictive marker, CPI

## Abstract

Recent studies reported that cytoplasmic dsDNA-induced activation of cyclic GMP-AMP synthase (cGAS)/stimulator of interferon genes (STING) signaling has tremendous potential for antitumor immunity by inducing the production of type I Interferon (IFN), resulting in activation of both innate and adaptive immunity. However, the potential role of STING signaling in modulating immunological checkpoint inhibitor (CPI) therapeutic efficacy remains unexplored. In this research, we employed the single-sample gene set enrichment analysis (ssGSEA) algorithm to calculate the enrichment score of STING signaling across 15 immunotherapy cohorts, including melanoma, lung, stomach, urothelial, and renal cancer. Logistic and Cox regression models were utilized to investigate the association between STING signaling and checkpoint inhibitor therapeutic response. Furthermore, we evaluated the tumor immunogenicity of STING1 molecule expression in the Cancer Genome Atlas (TCGA) pan-cancer datasets. STING signaling was associated with improved immune response in the Mariathasan2018_PD-L1, Gide2019_combined, Jung2019_PD-1/L1, and Gide2019_PD-1 datasets and with prolonged overall survival in the Gide2019_PD-1, Nathanson2017_post, Jung2019_PD-1/L1, and Mariathasan2018_PD-L1 datasets. However, the Braun_2020_PD-1 cohort exhibited worse prognosis outcomes in the high STING signaling subgroup. Our study extended the molecular knowledge of STING signaling activation in regulating the antitumor immune response and provided clinical clues about the combination treatments of STING agonists and CPIs for improving tumor therapeutic efficacy.

## Introduction

Checkpoint inhibitor (CPI) therapies, including antibodies targeting programmed cell death protein 1 (PD-1), programmed death-ligand 1 (PD-L1), or cytotoxic T-lymphocyte-associated protein 4 (CTLA4), have demonstrated astounding clinical efficacy in treating advanced cancers (Galon and Bruni, 2019). Analyses of clinical datasets have identified several positive predictive markers for CPI, including high levels of tumor mutation burden (TMB), PD-L1 overexpression, ARNT2 low expression, and T lymphocyte infiltration, among others (Litchfield et al., 2021). Recent studies reported that cytosolic DNA-sensing cyclic GMP-AMP synthase (cGAS)/stimulator of interferon genes (STING) signaling (referred to as STING signaling) has tremendous potential for antitumor immunity by inducing the production of type I Interferon (IFN) and chemokines and resulting in activation of both innate and adaptive immunity (Kwon and Bakhoum, 2020). However, the comprehensive evaluation of the STING signaling activities in pan-cancer and their potential role in modulating CPI therapeutic efficacy remains unexplored. In this study, we investigated the clinical implications of STING signaling in response to CPI treatment in 15 immunotherapy datasets across melanoma, lung, urothelial, stomach, and renal cancers and evaluated the tumor immunogenicity of STING1 expression among The Cancer Genome Atlas (TCGA) pan-cancer datasets.

## Method

We collated transcriptomic data for more than 900 CPI-treated patients and utilized standardized bioinformatics workflows and clinical outcome criteria to identify the role of cGAS/STING signaling in CPI sensitization (Supplementary Table S1). We validated the reliability of the ssGSEA-derived STING signaling score using integrated transcriptomic and phosphoproteomic datasets. The cGAS/STING-related gene set was curated from MSigDB V7.1 (REACTOME subset) and a literature review (Hopfner and Hornung, 2020) (Supplementary Table S2). The relative activity of STING signaling among individual CPI-treated tumors was quantified by using a single-sample GSEA (ssGSEA) algorithm with the GSVA package (Hanzelmann et al., 2013), which calculated separate enrichment scores for each pairing of a sample and a curated gene set. We also utilized two independent datasets (Gillette et al., 2020; Chen et al., 2020) with integrated transcriptomic and phosphoproteomic data to validate the reliability of enrichment scores on evaluation of STING signaling activities (Supplementary Method). A uniform clinical endpoint of response was defined across all the 15 CPI datasets derived from 11 independent studies based on the radiological response as per the RECIST criteria, with ‘‘CR/PR’’ being classified as a responder and ‘‘SD/PD,’’ as well as any ‘‘NE’’ cases, being classed as a non-responder. Logistic regression model and survival analyses were utilized to uncover the association between STING signaling and therapeutic response.

## Results

### Clinical implications of STING signaling in CPI immunotherapy

**FIGURE 1:**
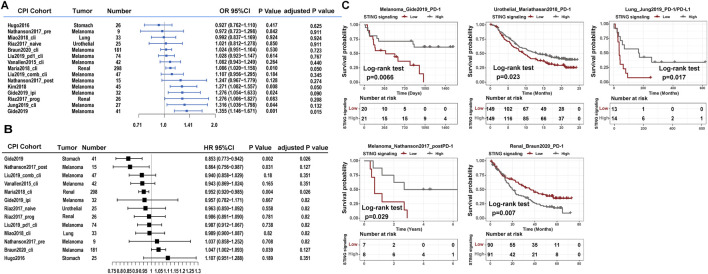
Clinical implications of STING signaling in CPI therapy. Forest plot representation of the association between the identified STING signaling and clinical response among 15 CPI datasets. **(A)** Logistic regression model estimated clinical immune response with STING signaling. **(B)** The Cox model estimated patients’ overall survival with STING signaling. The length of the horizontal line represented the 95% confidence interval for each subgroup. **(C)** Kaplan–Meier curves for overall survival of STING activity subtypes in CPI immunotherapy cohorts of the Gide2019_PD-1, Mariathasan2018_PD-L1, Jung2019_PD-1/L1, Nathanson2017_postPD-1, and Braun2020_PD-1 datasets.

The constructed STING signaling scoring scheme exhibited a promising correlation with the phosphorylation level of STING1, IRF3, and TBK1 in integrated transcriptomic and phosphoproteomic datasets (Supplementary Figures S1A–C). The ROC curve analysis also validated the predictive value of the established STING signaling scoring model (Supplementary Figures S1D, E). Furthermore, we adopted the model to explore the association of STING signaling with immunotherapy benefit and found that an improved immune response in the Mariathasan2018_PD-L1, Kim2018_PD-1, Gide2019_combined, Jung2019_PD-1/L1, and Gide2019_PD-1 datasets (logistic regression model, *P* < 0.05) and marginal significance in Riaz2017_progPD-1 (*P* = 0.083) (Figure 1A). Multivariate analysis indicated the association remained statistically significant in the Mariathasan2018_PD-L1, Gide2019_combined, Jung2019_PD-1/L1, and Gide2019_PD-1 datasets after considering age, gender, site, or stage (Supplementary Figures S2A–F). Although the association in the Kim2018_PD-1 dataset was not significant after multivariate adjustment, the STING signaling activities were significantly upregulated in Epstein–Barr virus (EBV)-positive, Microsatellite instability-high (MSI-H), and immune signature subtype (Supplementary Figures S3A–C). We also performed the survival analyses and noticed that STING signaling scores were significantly associated with prolonged overall survival in the Gide2019_PD-1, Nathanson2017_post, Jung2019_PD-1/L1, and Mariathasan2018_PD-L1 datasets (univariate Cox model, HR < 1, *P* < 0.05, Figure 1B). However, the STING signaling activity was inversely correlated with overall survival in the Braun2020_PD-1 dataset (HR, 1.047 [95% CI, 1.002 to 1.093], *P* = 0.039). Braun et al. demonstrated that numerous chromosomal alterations, rather than conventional genomic markers like TMB and CD8^+^ T cell infiltration, were associated with clinical responses or resistance to PD-1 blockade in advanced renal cell carcinoma. Leveraging these insights, we investigated these biomarkers and determined that STING signaling activity was inversely associated with the favorable PBRM1 mutation and purity and positively correlated with unfavorable chromosomal losses at 9q34.3 and 9q21.3 and ERV2282 overexpression (Supplementary Figures S3D–H). These findings further elucidate the unfavorable association between STING signaling activity and overall survival as observed in the Braun2020_PD-1 dataset.

We divided the four aforementioned datasets into low versus high expression subgroups based on the median STING signaling level. Prognosis analysis with the Kaplan–Meier model showed the comparable survival outcomes (log-rank test, *P* < 0.05; Figure 1C). Additionally, we explored the association between immune-related molecular characteristics and STING signaling score using the Mariathasan2018_PD-L1 and Jung2019_PD-1/L1 datasets, which provided sufficient sample size and molecular variables. Notably, the STING signaling activities were significantly upregulated in the immune-inflamed phenotype and higher neoantigen burden subgroups in Mariathasan2018_PD-L1 (Kruskal–Wallis test, *P* < 0.05; Supplementary Figures S4A, B) and were also significantly correlated with global methylation and aneuploidy levels in the Jung2019_PD-1/L1 dataset (Pearson correlation, *P* < 0.05, Supplementary Figures S4C, D).

### Association between STING signaling activities and identified predictors of immune response to CPI

**FIGURE 2:**
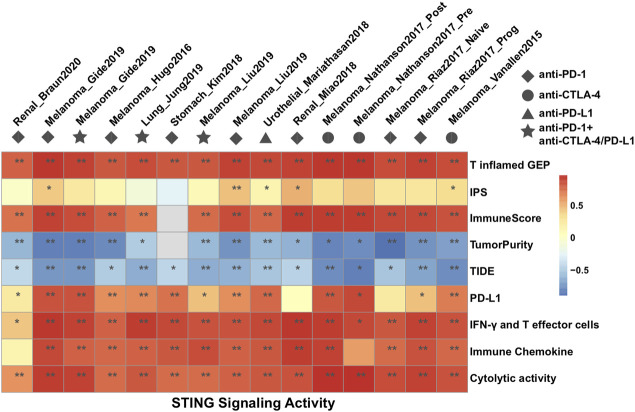
Heat maps showing the Spearman rank correlation coefficient between the identified STING signaling and predictors of immune response to CPI treatment among 15 datasets.

We further investigated the correlation of STING signaling activities with various transcriptomic signatures that had been proposed for predicting immune response to CPI therapy, including PD-L1, T inflamed-Gene expression profile (GEP), Immunophenoscore (IPS), ImmuneScore, Tumor Immune Dysfunction and Exclusion (TIDE), Interferon-γ (IFN-γ), and T effector cells, cytolytic activity, and immune chemokines (Supplementary Table S3). In most of the CPI datasets, T inflamed-GEP, ImmuneScore, TumorPurity, TIDE, IFN-γ, and T effector cells, and immune chemokines were strongly correlated with STING signaling activities, while the IPS signature was scarcely statistically significant (Spearman correlation; Figure 2). These findings suggested that STING signaling has a similar statistical significance to previously hypothesized predictors of CPI efficacy, and a prospective immunotherapy cohort and *in vivo* and *in vitro* experiments were required to validate the molecular mechanism of STING signaling on immune regulation.

### Tumor immunogenicity of STING1 in pan-cancer

**FIGURE 3:**
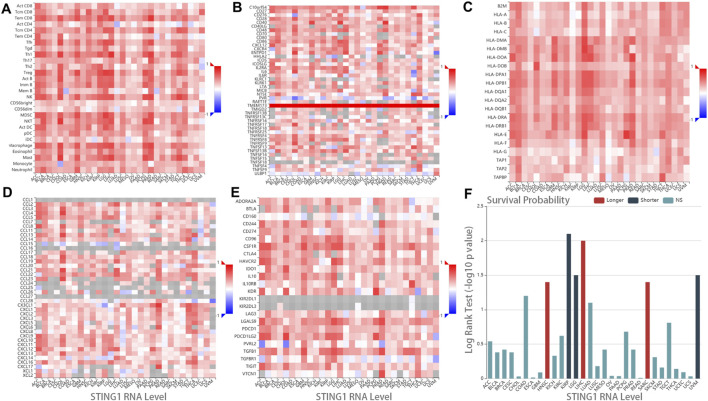
Molecular implications of STING1 expression in immunomodulation and prognosis. **(A–E)** Spearman’s correlation of STING1 RNA expression with immunogenomic features within the TCGA pan-cancer dataset, including **(A)** TILs, **(B)** immunostimulators, **(C)** MHCs, **(D)** chemokines, and **(E)** immunoinhibitors. **(F)** Associations between STING1 expression and overall survival across human cancers in the TCGA dataset. The red and dark-blue bars represent the STING1 expression significantly associated with longer and shorter survival, respectively. NS indicates not significant.

The tumor microenvironment (TME) has been associated with immune infiltration and response to immunotherapy across multiple cancer types (Fridman et al., 2017). Given the central role of STING1 (TMEM173) in STING signaling, we further investigated the correlation between STING1 RNA expression and key immunogenomic features, including tumor-infiltrating lymphocyte (TIL), immunoregulatory factors, major histocompatibility complex (MHC), and chemokines, across the TCGA pan-cancer datasets. The heatmap showed that STING1 was positively correlated with the abundance of multiple lymphocytes within solid tumors, such as activated CD8^+^ T cells, CD4^+^ T cells, dendritic cells (DC), macrophages, and natural killer (NK) cells (Figure 3A). Meanwhile, immunostimulators and MHC molecules were strongly associated with STING1 expression in a majority of cancer types (Figures 3B, C), suggesting that the activated STING signaling enforced tumor-antigen presentation and cross-primed CD8^+^ T cells for antitumor immunity (Zhang et al., 2020). In addition, STING1 was positively correlated with most inflammatory chemokines and checkpoint molecules (Figures 3D, E), further indicating combination treatment of STING1 agonists and CPIs can synergistically improve cancer biotherapeutic efficacy (Wang et al., 2020). We also investigated the association between STING1 expression and patient prognosis, as well as its differential expression in tumor versus normal tissues, using the TCGA pan-cancer dataset. We found that high STING1 expression was associated with worse survival outcomes in kidney renal papillary cell carcinoma (KIRP) and lower-grade glioma (LGG) (Figure 3F), suggesting that STING1 overexpression may serve as an unfavorable indicator of prognosis and CPI efficacy in renal carcinoma. Furthermore, STING1 expression was differentially regulated across various tumor types, with significantly higher levels observed in tumor tissues than paired normal tissues in kidney renal clear cell carcinoma (KIRC), pancreatic adenocarcinoma (PAAD), and thymoma (THYM), while lower expression was noted in KIRP, lung squamous cell carcinoma (LUSC), prostate adenocarcinoma (PRAD), uterine corpus endometrial carcinoma (UCEC), etc. (Supplementary Figure S5).

In summary, a comprehensive assessment of the STING signaling in CPI treatment will contribute to enhancing our understanding of innate immunity in CPI efficacy and guide the precision immunotherapy (Chen et al., 2022). In the upcoming era of combination or bispecific antibody immunotherapy (Zhang et al., 2023), our study extends the molecular knowledge of STING signaling activation in regulating the tumor immunogenicity and provides the clinical clues of the combination treatment of STING agonists and CPIs for improving tumor therapeutic efficacy. Further investigation in a prospective randomized clinical trial is warranted.

## Discussion

A comprehensive assessment of STING signaling in CPI treatment contributes to understanding innate immunity in CPI efficacy and guides precision immunotherapy. In the emerging era of combination or bispecific antibody immunotherapy, our study extends knowledge of STING signaling activation in regulating tumor immunogenicity. The findings suggest that combining STING agonists with CPIs may enhance the therapeutic efficacy. However, further investigation in prospective randomized clinical trials is warranted to validate these findings.

Recent advances in STING agonists for cancer immunotherapy are promising. Several, including TAK-676 (a CDN analog) and SNX281 (a non-CDN agonist), are in Phase I/II trials (Wang et al., 2020). Engineered bacteria like SYNB1891 are also being tested for direct, localized delivery of STING agonists, reducing side effects (Samson and Ablasser, 2022). Additionally, STING agonists combined with immune checkpoint inhibitors, such as ADU-S100 with spartalizumab, show promising results, advancing optimal treatment strategies (Chong et al., 2024; Hines et al., 2023).

However, several limitations must be considered. First, the study relies primarily on retrospective data from multiple immunotherapy cohorts, which can be subject to biases such as selection and recall bias, affecting the generalizability of the results. Second, the inclusion of different tumor types (e.g., melanoma, lung cancer, and urothelial carcinoma) introduces heterogeneity, making it difficult to draw universal conclusions regarding STING signaling efficacy across all cancer types. Additionally, the observed variability in the association between STING signaling and therapeutic response may be influenced by factors such as tumor microenvironment differences or genetic heterogeneity. The use of bioinformatics tools, like the ssGSEA algorithm, while valuable, is dependent on the quality and completeness of the data, and the results may not fully capture the complexities of the cGAS/STING pathway *in vivo*. Finally, while significant associations were found, prospective randomized clinical trials are needed to validate these findings, suggesting that the current results should be considered preliminary.

## Data Availability

The original contributions presented in the study are included in the article/Supplementary Material; further inquiries can be directed to the corresponding authors.

## Abbreviations

AA, Age acceleration; IBS, Irritable bowel syndrome; CI, Confidence interval; FEV1, Forced expiratory volume in one second; KDM-BA, Klemera–Doubal method biological age; SD, Standard deviation; SE, Standard error.
