# Supplementary material for: Clinical and molecular implications of cGAS/STING signaling in checkpoint inhibitor immunotherapy
Source: Front Mol Biosci. 2025 May 16;12:1556736. doi: 10.3389/fmolb.2025.1556736 (PMC12122296; doi:10.3389/fmolb.2025.1556736)
Supplement: Supplementary file 1 [file Supplementaryfile1.docx]

**Supplementary Information**

**Supplementary Method**

**Estimating the performance of STING signaling scoring model**

Active cGAS produces the cyclic GMP–AMP (cGAMP) and binds to stimulator of interferon genes (STING), leading to TANK-binding kinase 1 (TBK1)-dependent phosphorylation (P) of interferon regulatory factor 3 (IRF3) and STING([1](file:///E:\CHW\9-STING-letter\11-International%20Journal%20Of%20Surgery\Supplementary%20Method.docx#_ENREF_1)).The active IRF3 dimer translocates to the nucleus and activates transcription of type I interferon genes and adaptive immunity([2](file:///E:\CHW\9-STING-letter\11-International%20Journal%20Of%20Surgery\Supplementary%20Method.docx#_ENREF_2)). Considering the crucial role of phosphorylation of STING-TBK1-IRF3 on priming the STING pathway, we curated the phosphoproteomic dataset to identify the tumors with activated STING pathway and assessed the performance of RNA-based STING enrichment score. Briefly, lung cancer proteogenomic data of transcriptomic and phosphoproteomic were obtained from Gillette([3](file:///E:\CHW\9-STING-letter\11-International%20Journal%20Of%20Surgery\Supplementary%20Method.docx#_ENREF_3)) and chen et.al ([4](file:///E:\CHW\9-STING-letter\11-International%20Journal%20Of%20Surgery\Supplementary%20Method.docx#_ENREF_4)) dataset. We divided the tumors into high versus low phosphorylation levels of STING, TBK1 and IRF3, respectively, which by using the median phosphorylation as a cutoff point. Samples with both high levels of STING, TBK1 and IRF3 were recognized as the STING activated tumors. Transcriptomic RNA-seq based STING enrichment scores were compared with phosphorylation level of STING, TBK1 and IRF3. The receiver operating characteristic (ROC) curve was employed to assess the classification performance of the enrichment score and STING activation state.

**Reference:**

1. L. Sun, J. Wu, F. Du, X. Chen, Z. J. Chen. Cyclic GMP-AMP synthase is a cytosolic DNA sensor that activates the type I interferon pathway. *Science*. **339**, 786-91 (2013).

2. K. P. Hopfner, V. Hornung. Molecular mechanisms and cellular functions of cGAS-STING signalling. *Nat Rev Mol Cell Biol*. **21**, 501-21 (2020).

3. M. A. Gillette, S. Satpathy, S. Cao, S. M. Dhanasekaran, S. V. Vasaikar, K. Krug, F. Petralia, Y. Li, W. W. Liang, B. Reva, A. Krek, J. Ji, X. Song, W. Liu, R. Hong, L. Yao, L. Blumenberg, S. R. Savage, M. C. Wendl, B. Wen, K. Li, L. C. Tang, M. A. MacMullan, S. C. Avanessian, M. H. Kane, C. J. Newton, M. Cornwell, R. B. Kothadia, W. Ma, S. Yoo, R. Mannan, P. Vats, C. Kumar-Sinha, E. A. Kawaler, T. Omelchenko, A. Colaprico, Y. Geffen, Y. E. Maruvka, F. da Veiga Leprevost, M. Wiznerowicz, Z. H. Gumus, R. R. Veluswamy, G. Hostetter, D. I. Heiman, M. A. Wyczalkowski, T. Hiltke, M. Mesri, C. R. Kinsinger, E. S. Boja, G. S. Omenn, A. M. Chinnaiyan, H. Rodriguez, Q. K. Li, S. D. Jewell, M. Thiagarajan, G. Getz, B. Zhang, D. Fenyo, K. V. Ruggles, M. P. Cieslik, A. I. Robles, K. R. Clauser, R. Govindan, P. Wang, A. I. Nesvizhskii, L. Ding, D. R. Mani, S. A. Carr, C. Clinical Proteomic Tumor Analysis. Proteogenomic Characterization Reveals Therapeutic Vulnerabilities in Lung Adenocarcinoma. *Cell*. **182**, 200-25 e35 (2020).

4. Y. J. Chen, T. I. Roumeliotis, Y. H. Chang, C. T. Chen, C. L. Han, M. H. Lin, H. W. Chen, G. C. Chang, Y. L. Chang, C. T. Wu, M. W. Lin, M. S. Hsieh, Y. T. Wang, Y. R. Chen, I. Jonassen, F. Z. Ghavidel, Z. S. Lin, K. T. Lin, C. W. Chen, P. Y. Sheu, C. T. Hung, K. C. Huang, H. C. Yang, P. Y. Lin, T. C. Yen, Y. W. Lin, J. H. Wang, L. Raghav, C. Y. Lin, Y. S. Chen, P. S. Wu, C. T. Lai, S. H. Weng, K. Y. Su, W. H. Chang, P. Y. Tsai, A. I. Robles, H. Rodriguez, Y. J. Hsiao, W. H. Chang, T. Y. Sung, J. S. Chen, S. L. Yu, J. S. Choudhary, H. Y. Chen, P. C. Yang, Y. J. Chen. Proteogenomics of Non-smoking Lung Cancer in East Asia Delineates Molecular Signatures of Pathogenesis and Progression. *Cell*. **182**, 226-44 e17 (2020).

**Supplementary Figure S1-S5**

**
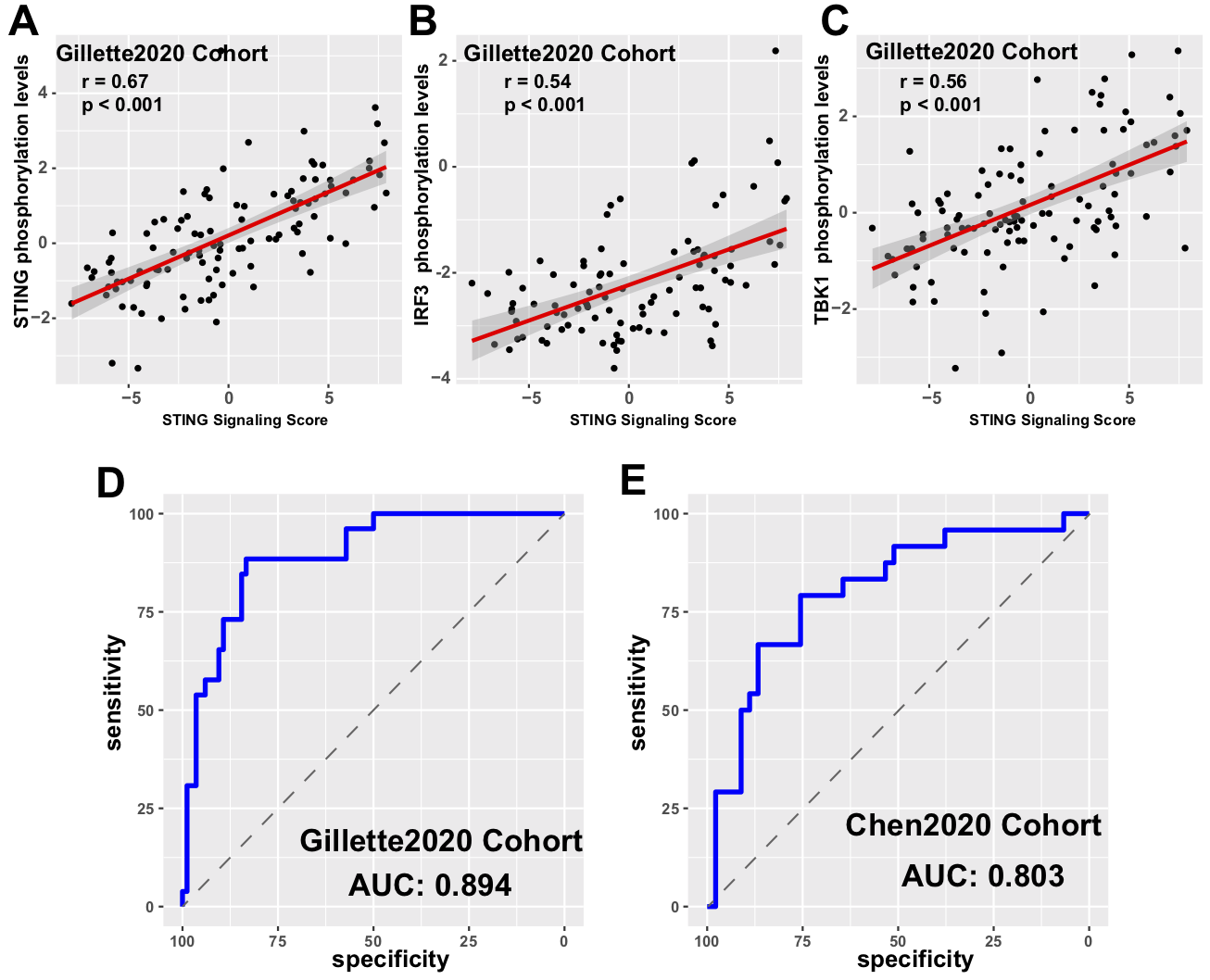
**

**Figure S1.** Evaluation of the performance of STING signaling scoring model in transcriptomic and phosphoproteomic datasets. Correlation of the phosphorylation levels of STING, IRF3 and TBK1 with STING signaling score in Gillette2020 cohort (A-C). The receiver operating characteristic (ROC) curve was used to assess the classification performance of the constructed STING score versus STING activate state in Gillette2020 and Chen2020 cohorts (D-E).


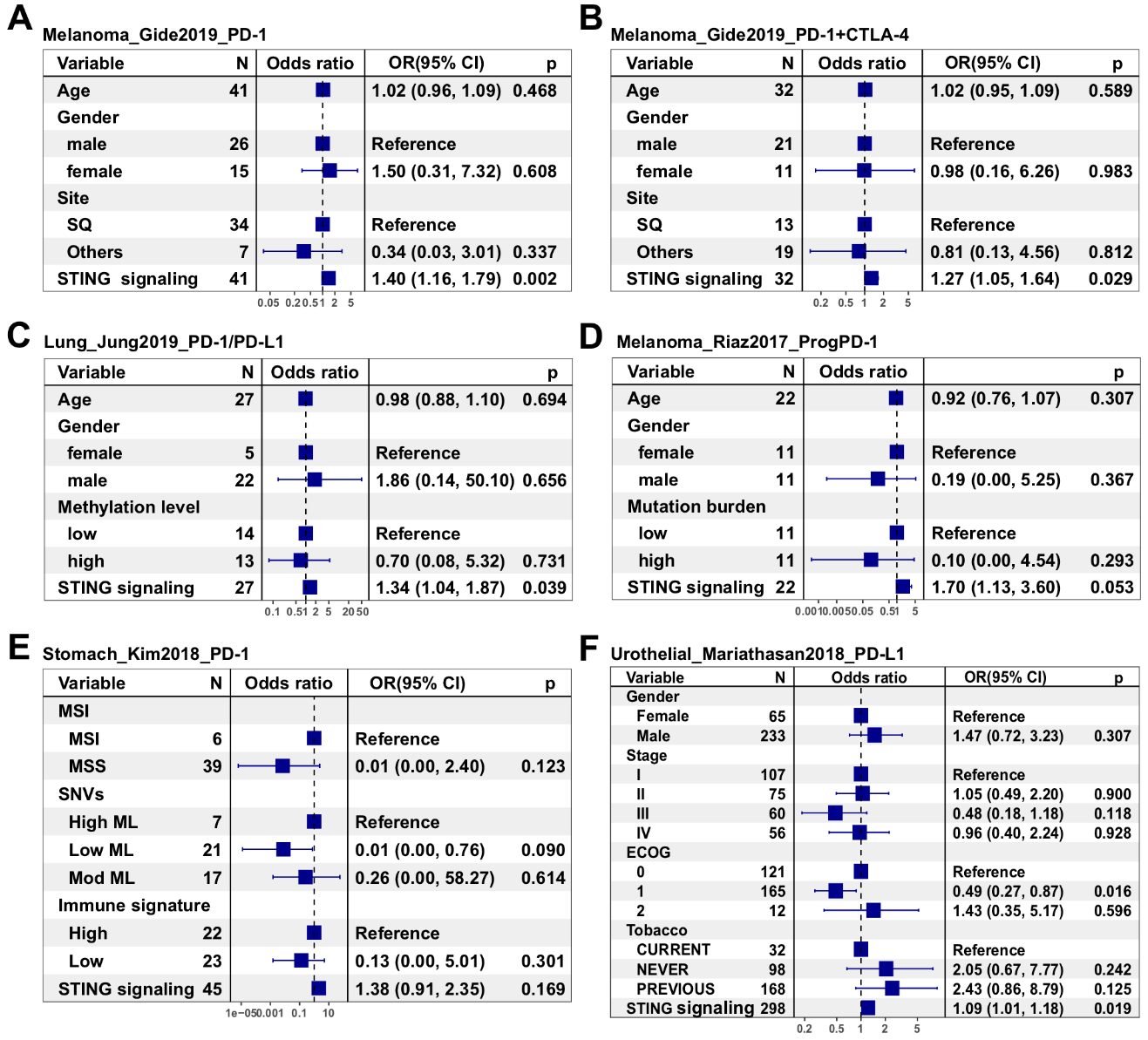


**Figure S2.** Forest plot representation of multivariate Logistic regression model depicting the association between the identified STING signaling and immune response in selected CPI datasets (A-F). The confounding factors include age, sex, site or stage variables.


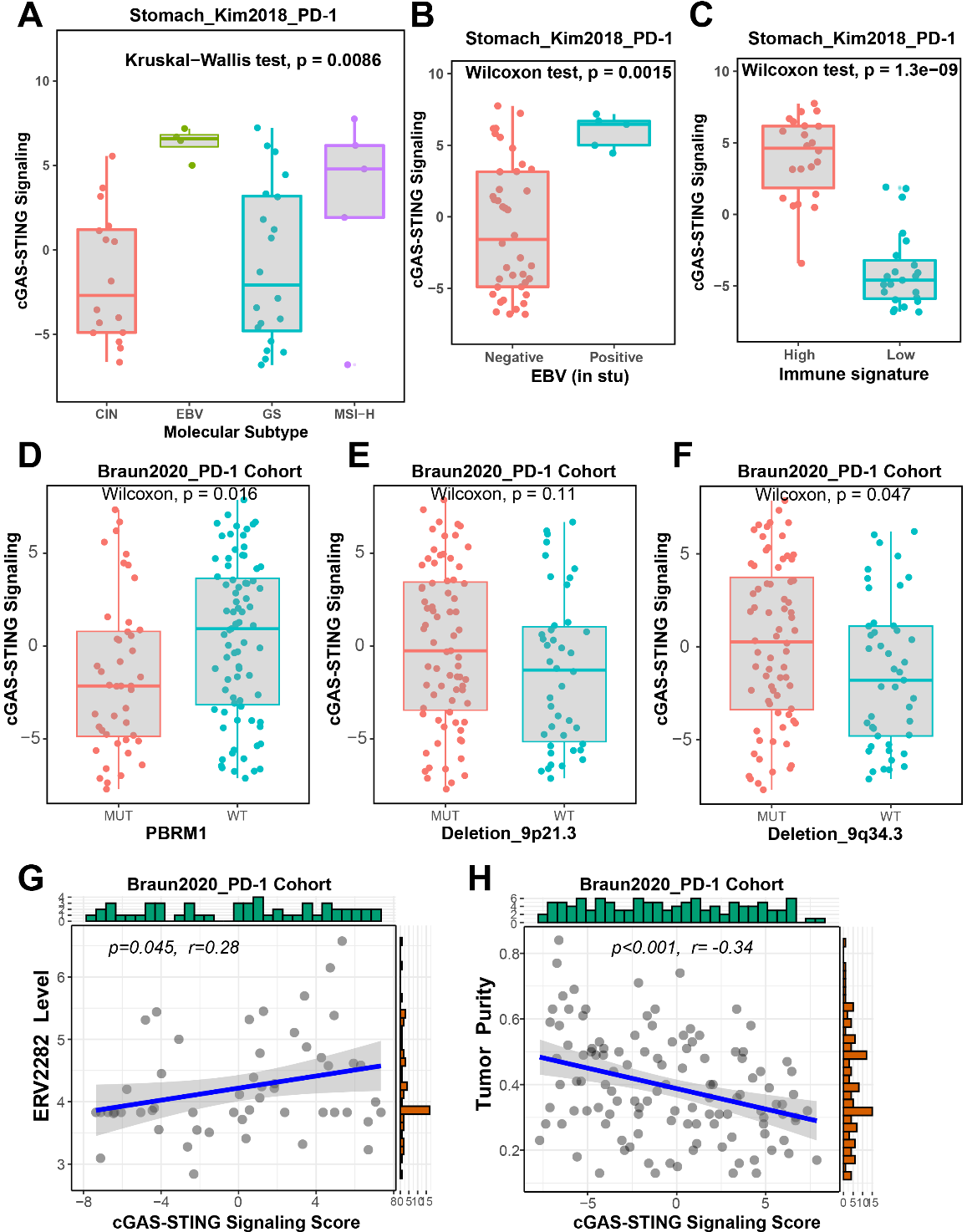


**Figure S3. Association between the cGAS-STING signaling and chromosomal alterations markers related to immune response or resistance.** Subgroup analysis of molecular phenotypes with identified STING singling in Kim2018_PD-1 cohort. The relative distribution of STING signaling activities was compared among different TCGA-stomach cancer subtype (A), EBV infection status (B) and Immune signature subtype (C). (D) STING signaling score were downregulated in the favorable PBRM1 mutation. (E-F) STING signaling score were upregulated in the unfavorable copy number alterations peaks of deletion on 9p21.3 and 9q34.3. (G-H) Correlation analysis revealed the ERV2282 and tumor purity was correlated with STING signaling score.


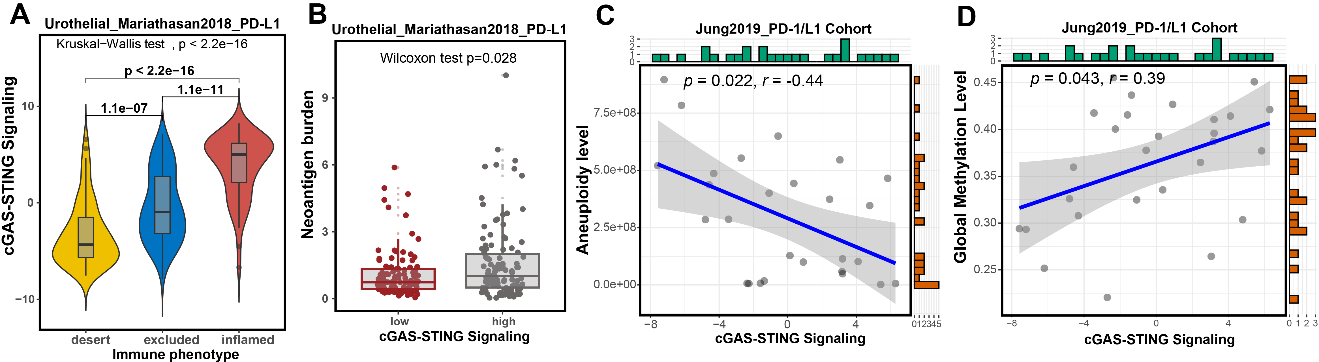


**Figure S4.** Subgroup analysis of molecular features with identified STING singling in Mariathasan2018_PD-L1 and Jung2019_PD-1/L1 cohort. (A)The relative distribution of STING signaling activities was compared among different Immune phenotypes. (B) Neoantigen burden was compared between high and low STING signaling subgroup. (C-D) Association between cGAS-STING signaling and aneuploidy and methylation level in Jung2019_PD-1/L1 cohort.


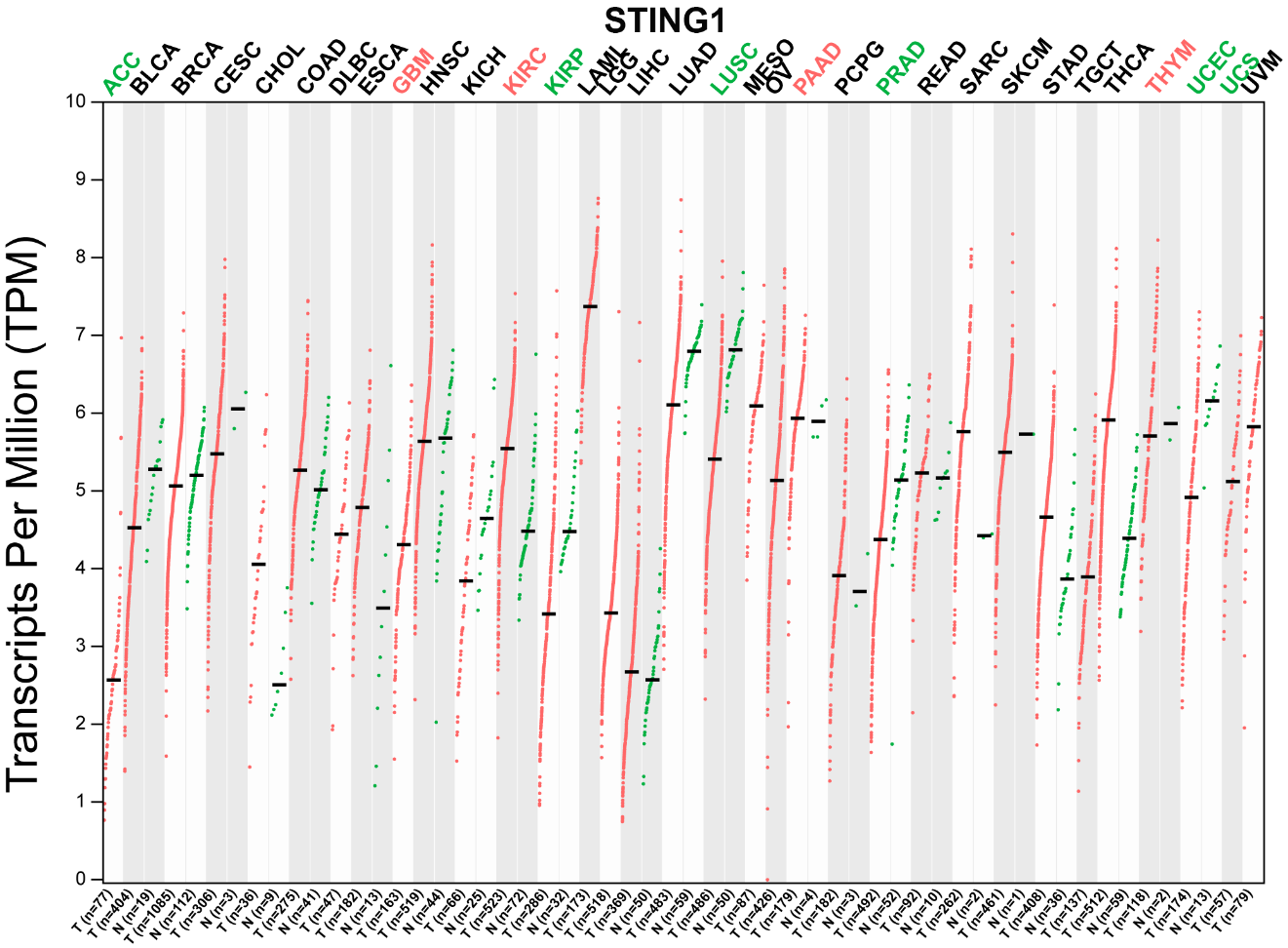


**Figure S5.** STING1 (TMEM173) expression profiles across all tumor samples and paired normal tissues in TCGA dataset. Each red dot and green dot represented the tumor tissues and paired normal tissues.

**Table S1.** Summary of the clinical characteristics of fifteen CPIs immunotherapy cohorts.

**Table S2.** List of the STING signaling-related genes.

**Table S3.** The curated predictors of transcriptomic signature that response to CPI immunotherapy.
